# Dynamic claw of dengue protease unveils druggability potential with high affinity allosteric inhibitors

**DOI:** 10.1093/pnasnexus/pgaf276

**Published:** 2025-11-04

**Authors:** Mrinmay Bhunia, Rajdip Misra, Anupam Maity, Sk Abdul Mohid, Shubham Kundu, Anirban Bhunia, Nakul C Maiti, Uttam Pal

**Affiliations:** Structural Biology and Bioinformatics Division, Indian Institute of Chemical Biology, Council of Scientific and Industrial Research, 4, Raja S.C. Mullick Road, Kolkata 700032, India; Academy of Scientific and Innovative Research (AcSIR), CSIR-Human Resource Development Centre, Ghaziabad, Uttar Pradesh 201002, India; Structural Biology and Bioinformatics Division, Indian Institute of Chemical Biology, Council of Scientific and Industrial Research, 4, Raja S.C. Mullick Road, Kolkata 700032, India; Structural Biology and Bioinformatics Division, Indian Institute of Chemical Biology, Council of Scientific and Industrial Research, 4, Raja S.C. Mullick Road, Kolkata 700032, India; Academy of Scientific and Innovative Research (AcSIR), CSIR-Human Resource Development Centre, Ghaziabad, Uttar Pradesh 201002, India; Department of Chemical Sciences, Unified Academic Campus, Bose Institute, EN-80, Sector V, Bidhan Nagar, Kolkata 700091, India; Structural Biology and Bioinformatics Division, Indian Institute of Chemical Biology, Council of Scientific and Industrial Research, 4, Raja S.C. Mullick Road, Kolkata 700032, India; Department of Chemical Sciences, Unified Academic Campus, Bose Institute, EN-80, Sector V, Bidhan Nagar, Kolkata 700091, India; Structural Biology and Bioinformatics Division, Indian Institute of Chemical Biology, Council of Scientific and Industrial Research, 4, Raja S.C. Mullick Road, Kolkata 700032, India; Academy of Scientific and Innovative Research (AcSIR), CSIR-Human Resource Development Centre, Ghaziabad, Uttar Pradesh 201002, India; Structural Biology and Bioinformatics Division, Indian Institute of Chemical Biology, Council of Scientific and Industrial Research, 4, Raja S.C. Mullick Road, Kolkata 700032, India

**Keywords:** loop dynamics, conformational switching, free energy landscape, enzyme activity, enhanced sampling

## Abstract

Several enzymes receive functional signals from allosteric site to the active site through conformational dynamics while domain dynamics can also play a significant role. Current investigation identifies a claw-like regulatory region near the active site of dengue protease, which remains in dynamic equilibrium as active (open) and inactive (closed) forms. Enhanced sampling with metadynamics showed that the flexibility of the T[RK][SN]G loop of the claw region is crucial for the protease activity as it helps in the opening of the claw-like structure near the active site, and allows the C-terminal hydrophilic part of NS2B cofactor to enter the claw and transform the protease into its active conformation. Binding kinetics and thermodynamics studies further reveal that allosteric modulator epigallocatechin-3-gallate (EGCG) binds to the enzyme in a biphasic manner and disrupts the active/inactive conformational dynamics and locks the enzyme in an inactive conformation. The inhibitor binding in this region introduces an energy barrier in the free energy landscape of NS2B cofactor binding. In the stronger mode, EGCG could directly bind into the claw, induces conformational changes, locking the dynamic loops in a closed position, preventing NS2B binding and rendering the enzyme inactive. Whereas in the weaker mode, EGCG binds to the T[RK][SN]G loop and perturbs its flexibility driving the claw structure into a more closed conformation and maintaining the protease in an inactive state. The understanding of the mechanism, therefore, would help researchers design more potent inhibitors targeting dengue protease and homologous enzymes.

Significance Statement This study addresses the significant challenge of designing inhibitors for the dengue NS2B/NS3 serine protease, which is complicated by its shallow active site and the presence of charged residues around it. Known allosteric inhibitors show low potency due to limited understanding of its allosteric regulation mechanisms. Here, we established the conformational and domain dynamics of the protease and demonstrated how the inhibitor modulates these dynamics by binding to a specific allosteric site. This work established the allosteric inhibition mechanism of dengue protease function and suggests a new direction for drug design against this protease family, proposing that covalent molecules could be designed to irreversibly lock the claw region and inactivate the protease.

## Introduction

Proteins, functioning as molecular machines, support all biochemical processes of cells (1). To perform these functions, protein possess dynamic structures comprising various conformational states referred to as conformational ensembles that rapidly interchanges between slightly active and inactive forms (2). The precise regulation of the interconversion process ensures that these conformational states coexist in the appropriate proportions necessary for biological function, ligand binding, and allostery (3–6). Allostery or allosteric regulation is a phenomenon in which functional activity of protein is altered by binding of a allosteric modulator at a site that is topologically distinct from the active site. It is considered the “second secret of life” because it is the most direct and efficient way to regulate protein function, signal transduction, enzyme catalysis, transcriptional and translational regulation (7). Allosteric modulators modulate protein's function allosterically by affecting not only thermodynamics properties but also conformational dynamics of protein (8). Allostery does not create new conformation of protein, they only perturbate the relative distribution of conformations that are already present within the protein (3, 9). Conformational dynamics serve as conduits that transmit signal from allosteric site to orthosteric site, and thus affects the binding of orthosteric ligands. Dysfunction of allosteric communication between allosteric and orthosteric sites by mutation associated with several types of human diseases (3, 10, 11). Understanding the principles of allostery, along with the conformational dynamics and thermodynamics involved, has paved the way for the design of potent allosteric drugs. These drugs present several advantages over traditional orthosteric inhibitors, primarily by minimizing off-target effects. Furthermore, allosteric inhibitors do not need to compete with the natural substrate at the active site, which is particularly beneficial when the substrate concentration is high. Consequently, there is a burgeoning interest in exploring how allosteric modulators influence protein function and how this mechanism can be utilized in the development of new therapeutics aimed at a wide range of diseases.

In this study, we utilized the dengue NS2B/NS3 protease (NS2B/NS3pro) and epigallocatechin-3-gallate (EGCG) as a model system to investigate the mechanisms of allosteric regulation from the perspectives of conformational dynamics and thermodynamics. This enzyme is a critical drug target for dengue virus and other similar flaviviruses, including Zika virus, West Nile virus, and yellow fever virus. Till date effective antiviral drugs or vaccines are not available to combat these diseases. Similar to other flaviviruses, the dengue viral NS2B/NS3 protease plays a crucial role in viral replication and the maturation process by cleaving the viral polyprotein at the junctions of NS2A/NS2B, NS2B/NS3, NS3/NS4A, and NS4B/NS5, as well as within the capsid protein (12–16). This cleavage is essential for the release and maturation of individual structural and nonstructural proteins. Active-site inhibitor design of the protease faced challenges due to the relatively shallow active site and the presence of charge residues surrounding it (17–22). To address this allosteric inhibitor design is essential to inhibit the protease. Elucidating the mechanistic insights of allosteric regulation of the protease is crucial for the rational design of allosteric inhibitors targeting this enzyme.

**Scheme 1.**
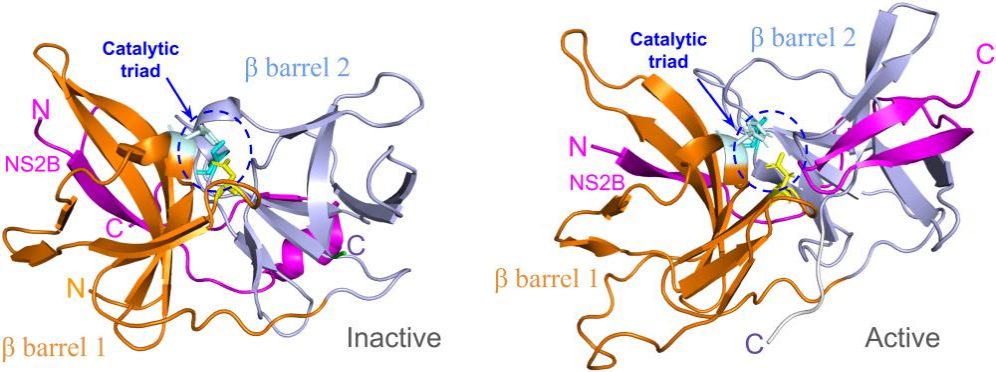
Structure of NS2B/NS3pro. The two β-barrels, the active site and the position of NS2B in the inactive and active conformations are shown.

Dengue NS3 is a multifunctional protein consisting of two domains: the N-terminal 185 amino acid residues long serine protease domain (NS3pro) and the C-terminal helicase domain with nucleoside triphosphatase activity (23–25). NS3pro (Scheme 1) has a trypsin-like fold with two β barrels each with six β strands and a catalytic triad of His51, Asp75, and Ser135 situated in the center of two β barrels (26, 27). Active site of the enzyme forming a pocket-like structure which is enclosed by two beta turns and flanked by antiparallel beta-sheets (22). One of these turns consists of the hydrophobic amino acid residues (GI[IF]G) while the other comprises the hydrophilic amino acid residues (T[RK][SN]G) (28). The central hydrophilic segment in NS2B is crucial for the NS3 protease's activity, in switching between inactive and active conformations (18, 22, 25, 26, 29–31). The N-terminal portion (residue range 50–62) of NS2B remains connected to the N-terminal beta barrel of NS3pro and in the inactive form, the C-terminal part remains solvent exposed, while in the active form, it wraps around the core of NS3pro and enters a deep cleft close to the active site.

The conformational dynamic interplay between active and inactive states is crucial for the enzyme's functionality. Perturbation of these conformational dynamics can inhibit enzyme activity, making it an important approach for drug design. Allosteric regulation shifts the relative distribution of enzyme conformations, thereby affecting conformational dynamics. Currently, numerous studies are investigating allosteric inhibitors targeting the NS2B-NS3 protease (18, 26, 31–34). Among the noteworthy discoveries is EGCG, a natural compound reported to exert allosteric inhibition on the enzyme (32). We selected this molecule to investigate the mechanism behind the allosteric inhibition of the dengue NS2B/NS3 protease. Through a combination of biophysical techniques, molecular dynamic, and enhanced sampling with metadynamics analysis, we unveiled the mechanistic pathway of allosteric inhibition. This knowledge holds significant promise for rational drug designs targeting the dengue protease.

## Results

### Biphasic binding of EGCG with NS2B/NS3pro and allosteric modulation of enzymatic activity

**Fig. 1.**
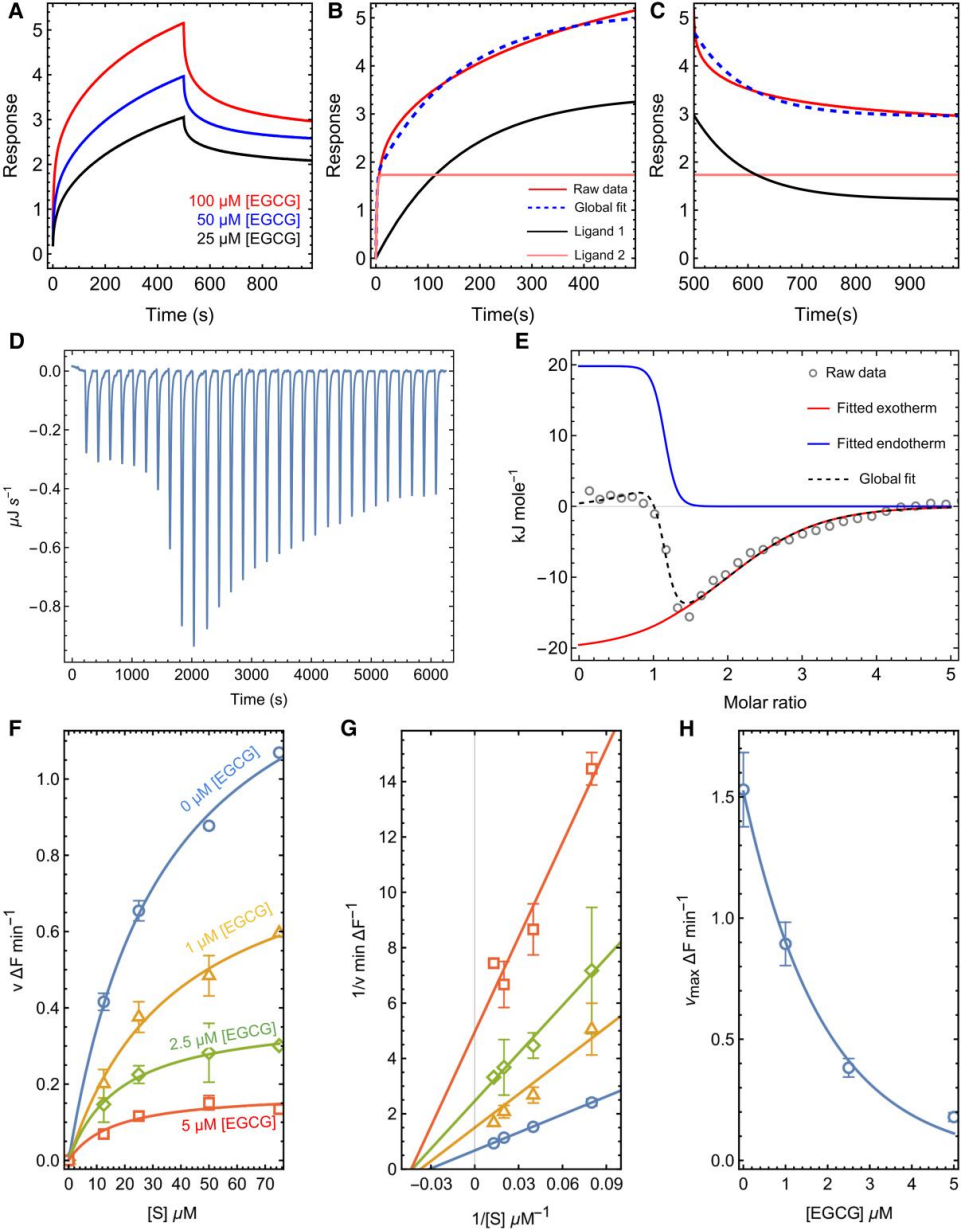
EGCG binds with dengue NS2B/NS3pro: A) Biolayer interferometry sensorgrams depicting the binding of EGCG with dengue NS2B/NS3pro at three concentrations: 100 μm, 50 μm, and 25 μM. The first 500 s represent the association phase, followed by the dissociation phase in the last 500 s. B, C) Deconvolution of the association and dissociation phases for 100 μM EGCG binding kinetics by fitting the data in 2:1 model fitting equation. One EGCG binds with the protease with first association and slow dissociation and the second EGCG binds with slow association and first dissociation rate. D) ITC measurement of the corrected heat rate (μJ/s) for NS2B/NS3pro titrated with EGCG. E) ITC data were fitted using biphasic binding equations to calculate thermodynamic parameters. The open circles represent the heat change at various molar ratios of EGCG and NS2B/NS3pro. The solid lines corresponds to the exothermic and the endothermic binding fits, and the dotted line represents the Global fit of both exothermic and endothermic processes. Data shows that one EGCG binds with protease with endothermic reaction and other with exothermic process. F) Michaelis–Menten plot showing the velocity of the enzyme reaction at varying substrate concentrations and varying EGCG concentrations, fitted with the Michaelis–Menten equation to determine kinetic parameters. G) Lineweaver–Burk plot, a double-reciprocal plot demonstrates that the *K_m_* value remains constant while the *v*_max_ value varies with different EGCG concentrations indicating that EGCG inhibits NS2B/NS3pro in a noncompetitive manner. H) *v*_max_ versus EGCG concentration plot to determine IC50 value, depicting 1.33 μM concentration of EGCG required to reduce NS2B/NS3pro activity by 50%.

Bio-layer interferometry (BLI) was employed to quantify the binding interactions between EGCG and the NS2B/NS3 protease complex. BLI, an optical technique that detects shifts in the interference pattern of white light reflected off the biosensor surface and measures changes in optical thickness due to the ligand immobilization process. This method enabled us to determine key binding parameters, including the association rate constant (*k_a_*), dissociation rate constant (*k_d_*), equilibrium dissociation constant (*K_D_*), and binding stoichiometry between EGCG and NS2B/NS3pro. Figure 1A illustrates the BLI response curves as a function of time with increasing EGCG concentrations (25, 50, and 100 μM). The initial 500 s kinetics reflects the association phase of EGCG binding to NS2B/NS3pro, followed by the dissociation phase (501–1000 s). The binding data did not fit a 1:1 binding model (Fig. S1); however, it closely fits with a 2:1 association and dissociation model, indicating a binding stoichiometry of 2:1. Figures 1B and C and S2 show the deconvolution of biphasic binding kinetics in the association and dissociation phases for EGCG binding kinetics. The data suggest that one EGCG molecule binds the protease much strongly with a first association rate constant (*k_a2_*) 0.536 M^−1^ s^−1^. This molecule remains bound during the wash phase. The second EGCG molecule binds weakly to the protease with a slower association rate constant (*k_a1_*) 0.006 M^−1^ s^−1^, and it dissociates during the wash phase with a dissociation rate constant of (*k_d1_*) 0.011 M^−1^ s^−1^.

In order to obtain binding thermodynamics, isothermal calorimetry (ITC) was performed, which measures the heat released or absorbed during the biochemical process under constant temperature conditions (35, 36). Figure 1D shows the microjoule heat changes that occur as EGCG is added stepwise to the NS2B/NS3pro solution over time. The binding free energy and stoichiometry of EGCG interaction with NS2B/NS3pro were determined by fitting the data to a biphasic isothermal equation (Fig. 1E). The biphasic isotherm displays two clear phases: an initial fast endothermic process followed by a subsequent slow exothermic process, both occurring at constant temperature. Stoichiometry (*n*) for endothermic reaction is ∼1 whereas that of exothermic reaction is ∼2. The enthalpy change (Δ*H*) for the two phases were 19.81 and −20.16 kJ/mole, respectively. Equilibrium constant for the endothermic process is 5.90 × 10^7^ M^−1^ and for the exothermic process 8.12 × 10^6^ M^−1^. Binding free energy (Δ*G*) for endothermic and exothermic processes was determined to be −7.42 and −6.60 kJ/mol, respectively. The ITC results demonstrate that the fast endothermic binding event is likely due to a conformational change in the enzyme upon ligand interaction, while the slower exothermic binding event may reflect the stabilization of the enzyme–ligand complex.

In vitro enzyme inhibition assay was conducted to study whether binding EGCG to NS2B/NS3pro interferes with the activity of the enzyme. By varying the inhibitor concentration and substrate concentration, enzyme activity was measured and fitted with the Michaelis–Menten enzyme kinetics equation to obtain the maximum velocity (*v*_max_) and Michaelis constant (*K*_M_) (Fig. 1F). The *v*_max_ decreased with increasing concentration of EGCG. Double reciprocal plot (Fig. 1G) linearized Michaelis–Menten data to show the trends in *K*_M_ and *v*_max_. The plot shows that *K*_M_ value remains unaltered but *v*_max_ gradually decreased with increasing EGCG, indicating allosteric nature of the inhibition. A inhibitor concentration versus *v*_max_ plot (Fig. 1H) gave the inhibitory constant (IC_50_), which is the concentration of the inhibitor at which maximum enzymatic velocity is reduced by 50%. IC_50_ for EGCG was found to be 1.33 μM.

Saturation transfer difference (STD) NMR was performed to identify the functional groups of EGCG that interact with NS2B/NS3pro at an atomic level. It is a technique used to study interactions between small molecules and larger biomolecules by observing the transfer of magnetization from the saturated protons of the larger molecule to the bound ligand (37, 38). The EGCG to NS2B/NS3pro concentration was kept at a 100:1 ratio in this assay to increase the “STD amplification factor.” A careful examination of the STD data reveals that the aromatic ring proton groups H2″ and H6″ from the B″ aromatic group interact substantially with the NS2B/NS3pro protein, as demonstrated by the highest STD peaks of the aromatic ring proton groups (Fig. S3). The H2′ and H6′ protons from the B″ aromatic ring are likewise near to the NS2B/NS3pro, as evidenced by a 64% saturation transfer from the protein to the B″ ring protons. The H6 and H8 protons, on the other hand, produced extremely faint STD signals (19%), suggesting the ligand protons distance from the protein molecule.

### Effect of EGCG binding on the structure and dynamics of NS2B/NS3pro

BLI, ITC, STD NMR, and in vitro enzyme inhibition assays together demonstrated that EGCG binds to and inhibits dengue NS2B/NS3pro allosterically. However, the question remains: does EGCG interfere with protease activity by altering the structure or dynamics of the protease? To investigate, circular dichroism (CD) spectroscopy was employed to monitor structural alterations in NS2B/NS3pro due to EGCG binding. CD spectroscopy is an optical technique that provides information about the secondary structure of proteins by measuring the differential absorption of left- and right-handed circularly polarized light (39). Figure S4 shows CD spectra of NS2B/NS3pro with increasing concentration of EGCG. Secondary structural compositions were obtained by deconvolution of the CD spectra and shown as a function of EGCG concentration (Fig. S4). No significant secondary structural changes were observed upon EGCG binding indicating dynamics of NS2B/NS3pro could be the determining factor of its activity.

**Fig. 2.**
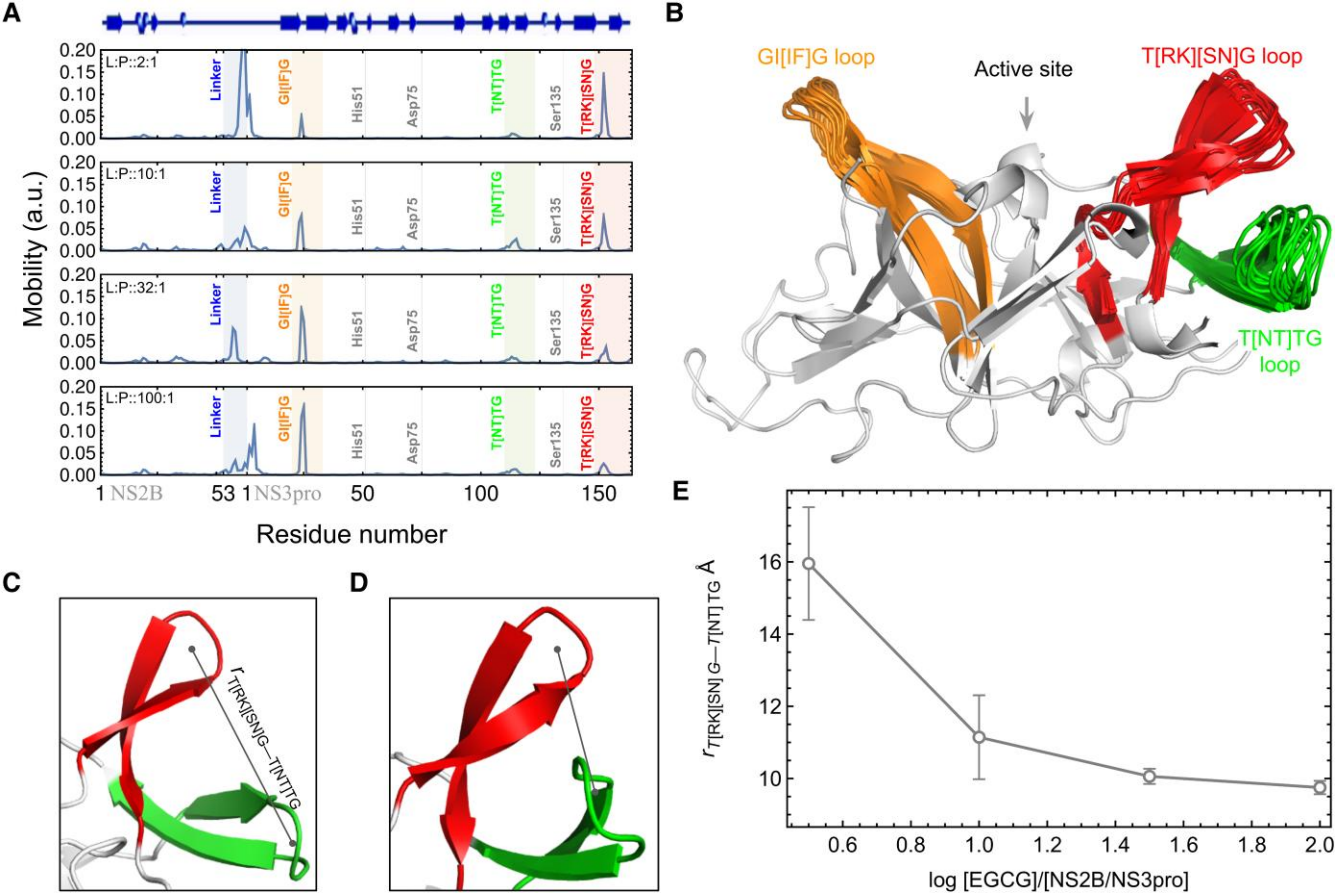
Mobility of NS2B/NS3pro in presence of EGCG. A) Mobility plots of the protease at various EGCG molar ratios, showing a gradual decrease in the motion of the T[RK][SN]G loop with increasing EGCG concentration. B) Range of motion for three loop regions (GI[IF]G, T[NT]TG, and T[RK][SN]G) of the protease as observed in MD simulations. C, D) Equilibrium structure of T[NT]TG and T[RK][SN]G loop of dengue NS2B/NS3pro at EGCG to NS2B/NS3pro mole ratio 2:1 and 100:1, respectively showing that two loop comes closer at mole ratio 100:1. E) Distance measurements between the T[NT]TG and T[RK][SN]G loops at different log of molar ratios of EGCG to NS2B/NS3pro, showing a gradual decrease in distance, which indicates cleft closure.

Loops structure and their dynamics have received significant attention in recent years for their role in understanding enzyme function, the mechanisms of allosteric regulation and their importance in the design of new enzymes. The most ubiquitous loop motions in many natural enzymes, such as triosephosphate isomerase (40), dihydrofolate reductase (41), protein tyrosine phosphatase (42), or orotidine 5′-monophosphate decarboxylase (43), are some well-known examples involving the closing and opening of activation loops around the active site. Our earlier report also observed a similar kind of loop motion in dengue NS2B/NS3 serine protease (28). The NS2B/NS3pro active site features a flat structure surrounded by two hairpin-like supersecondary structures (beta-turn-beta): one with a hydrophobic GI[IF]G loop and the other with a hydrophilic T[RK][SN]G loop. The T[RK][SN]G loop of NS2B/NS3pro shows a correlated motion which is linked to its activity (28). By modulating these motions the activity could be modulated, which is specifically important for allosteric regulation. To study the correlated dynamics in presence of EGCG, we have performed a series of molecular dynamics simulations of NS2B/NS3pro with increasing molar ratios of EGCG. Figure 2A shows the residue-wise mobility plots of NS2B/NS3pro with different molar ratios of EGCG. We have found that the motion of the T[RK][SN]G loop region reduces significantly with the increasing molar ratios of EGCG indicating that the structure is becoming more rigid. Figure 2B shows the native structure of NS2B/NS3pro, highlighting the range of motion in three loop regions: GI[IF]G, T[NT]TG, and T[RK][SN]G. When compared the equilibrium structures of NS2B/NS3pro at EGCG to NS2B/NS3pro molar ratios of 2:1 and 100:1 (Fig. 2C and D, respectively), the T[NT]TG and T[RK][SN]G loops were found to move closer. Figure 2E shows measurements of loop distances at different molar ratios of EGCG to NS2B/NS3pro indicating that the T[NT]TG and T[RK][SN]G loops gradually approached each other. These conformational changes in the cleft region, induced by EGCG, may play a key role in its mechanism of action.

### Inactive and active conformational switching of NS3pro and NS2B binding

**Fig. 3.**
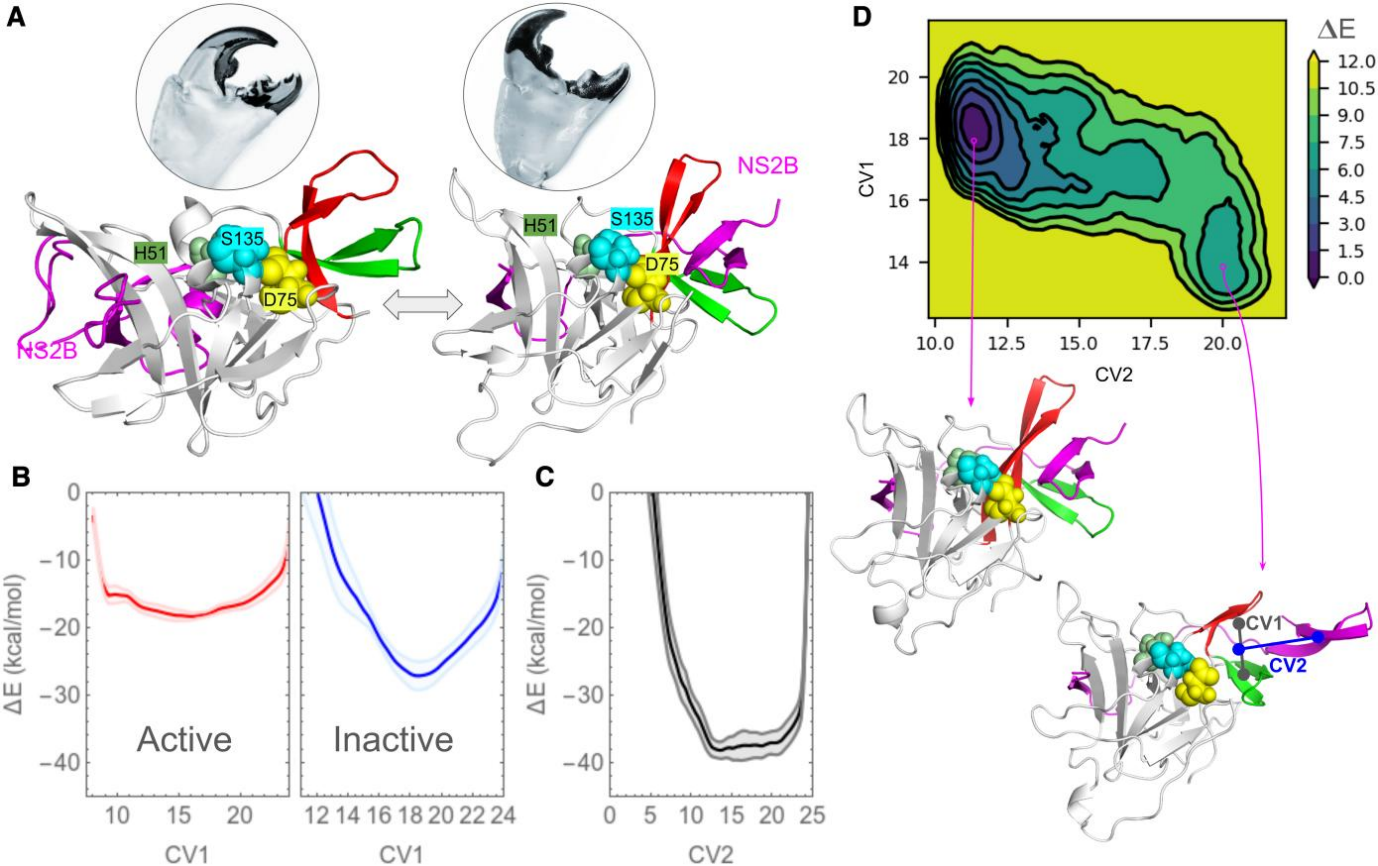
NS2B binding dynamics with NS3 protease. A) Active and inactive transformation of protease analogous to crab claw opening and closing indicates that motion of T[RK][SN]Gl loop required for claw opening and NS2B binding in active conformation. Open and closed form of crab claw, represent active, and inactive form the protease. B) Free energy landscape of the claw motion in NS2B bound and unbound states. C) Free energy profile of NS2B binding and dissociation into the claw region. D) A representative 2D free energy surface of NS2B binding to the claw region obtained by perturbation of two collective variables: CV1 along the center of mass distance between the two loops of the claw and CV2 along the center of mass distance between the claw and the NS2B C-terminal loop. See Fig. S11 for all the replicas. Protein conformations at the two different minima on the energy landscape representing the active and inactive form of dengue NS2B/NS3pro are shown.

Molecular dynamics mobility studies have demonstrated that EGCG inhibits the mobility of the T[RK][SN]G loop, thereby suppressing enzyme activity. This prompts the question: why does inhibiting the mobility of the T[RK][SN]G loop correlate with enzyme activity inhibition? To understand the event, it is crucial to conduct energy profiling of conformational dynamics of NS2B/NS3pro. The NS2B/NS3pro complex adopts an active conformation, with the C-terminal end of NS2B positioned in the deep cleft of NS3pro resembling a crab claw near the active site constituted by the T[NT]TG and T[RK][SN]G loops as shown in Fig. 3A. Similar to a claw of a crab, T[NT]TG and T[RK][SN]G loop opens up to hold NS2B. Thus, the conformational switching of this claw between open and closed conformation is crucial for NS2B binding and its activity (Fig. 3A). We performed metadynamics simulations to explore the free energy landscape of active and inactive conformation switching. Metadynamics enhance sampling of rare events by adding biasing potential, which helps to explore and reconstruct free energy surfaces efficiently. In Fig. 3B, the plot of free energy versus the center of mass distance of T[NT]TG and T[RK][SN]G loop (CV1) demonstrates that the closed-claw conformation exhibits an energy minimum near 15 Å in the free energy profile, whereas the open-claw conformation (NS2B bound) shows a minimum near 18 Å. Interestingly we observed that the free energy curve of closed-claw (inactive) conformation is wider than open-claw conformation which indicates that inactive conformation exhibits greater flexibility than the NS2B bound (active) conformation. We further conducted metadynamics analysis to explore NS2B association and dissociation energy profile with the claw-like structure of T[NT]TG and T[RK][SN]G loop (Fig. 3C). Center of mass distance between the claw-like structure of NS3pro and C-terminal hydrophilic part of NS2B (CV2) versus free energy plot (Fig. 3C) showed that there is no significant energy barrier in NS2B association and dissociation. Then both the CV1 and CV2 were perturbed simultaneously, to obtain a the free energy landscape of NS2B association and dissociation (CV2) along with claw opening and closing (CV1). Figure 3D shows two distinct energy minima in the free energy landscape: the more negative one appears near CV1 ≈ 18 Å (open-claw) and CV2 ≈ 11 Å (NS2B bound) and the other minimum appears near CV1 ≈ 14 Å (closed-claw) and CV2 ≈ 20 Å (NS2B unbound), which indicate that active conformation is energetically more favorable than the inactive conformation, however, there is a finite energy barrier which is affected by the claw dynamics.

### Inhibition of NS2B binding by EGCG

**Fig. 4.**
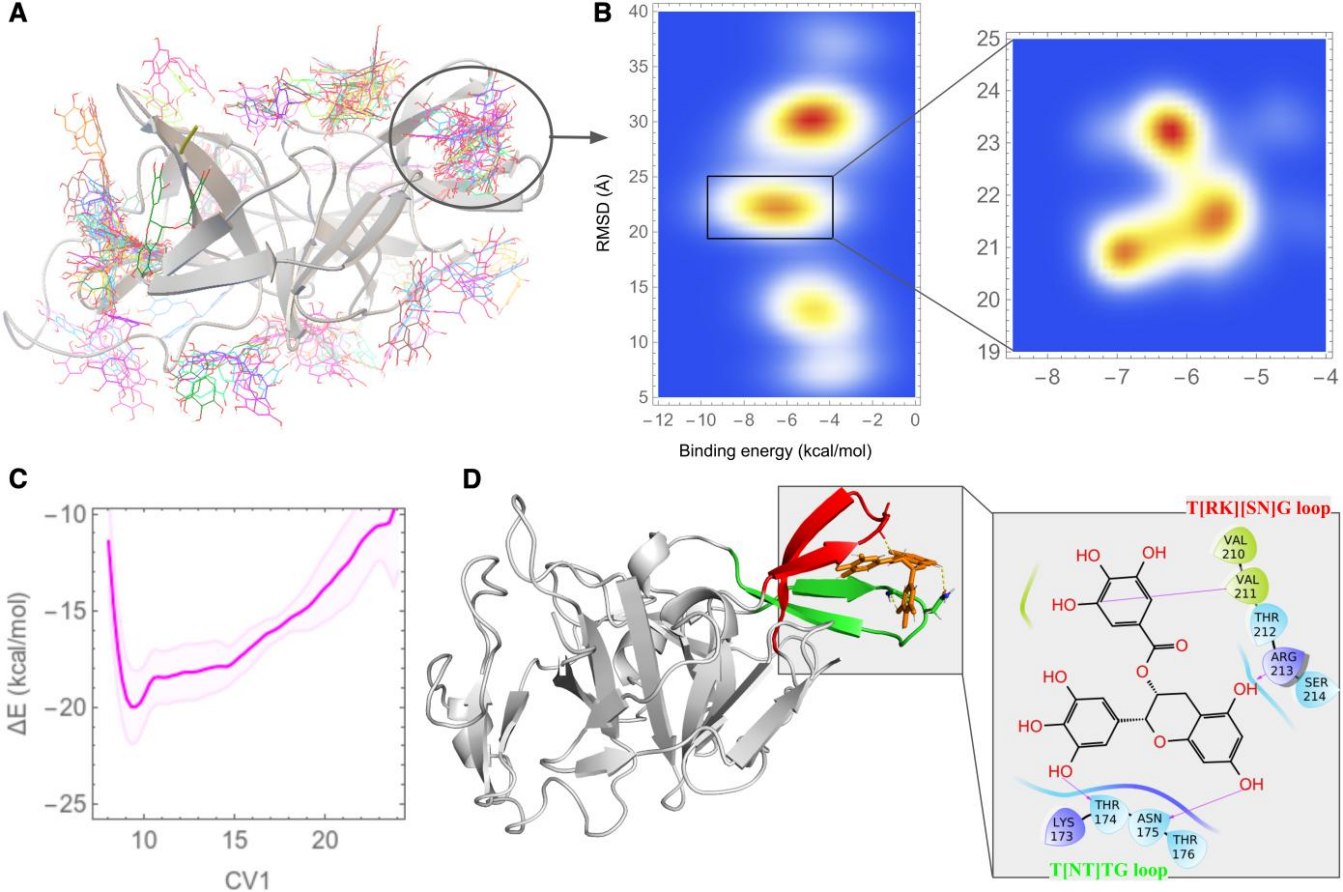
Binding site of EGCG on NS3 protease and the effect of EGCG on conformational dynamics of the claw structure. A) Blind docking results superimposed. B) Clustering of docked conformations on a free energy surface. C) Free energy profile of claw dynamics when bound to EGCG as obtained from metadynamics simulations. D) Structural snapshot EGCG bound closed claw structure of protease.

To sample the best possible binding regions of EGCG on the NS2B/NS3pro, blind docking simulations were performed and the specific protein site were identified where EGCG can bind with high affinity. Figure 4A depicts a snapshot of the docked-protein structure, while Fig. 4B illustrate the clustering of docked conformations on a free energy surface, demonstrating that EGCG exhibits the lowest binding energy in the claw-like structure of NS2B/NS3pro.

Lowest energy EGCG-bound NS2B/NS3pro structure was subjected to further metadynamics analysis to investigate the free energy profile of claw motion under the EGCG-bound condition. As depicted in Fig. 4C, EGCG binding results in a shift of the energy minima in the free energy profile of the claw-like structure of T[NT]TG and the T[RK][SN]G loops to 9 Å (a tightly closed-claw conformation) from 15 Å in the NS2B unbound closed-claw conformation of the protein. The observed shift also accompanies with a narrow and more negative peak profile compared to the shallow and wide peak profile in the claw dynamics of NS2B or EGCG unbound form. The narrower free energy profile upon EGCG binding also indicates a reduction in the flexibility of the claw. Figure 4D presents snapshots of the EGCG-bound, more closed conformation of the claw structure. The figure illustrates that EGCG interacts with both the T[NT]TG and T[RK][SN]G loops within the claw structure.

**Fig. 5.**
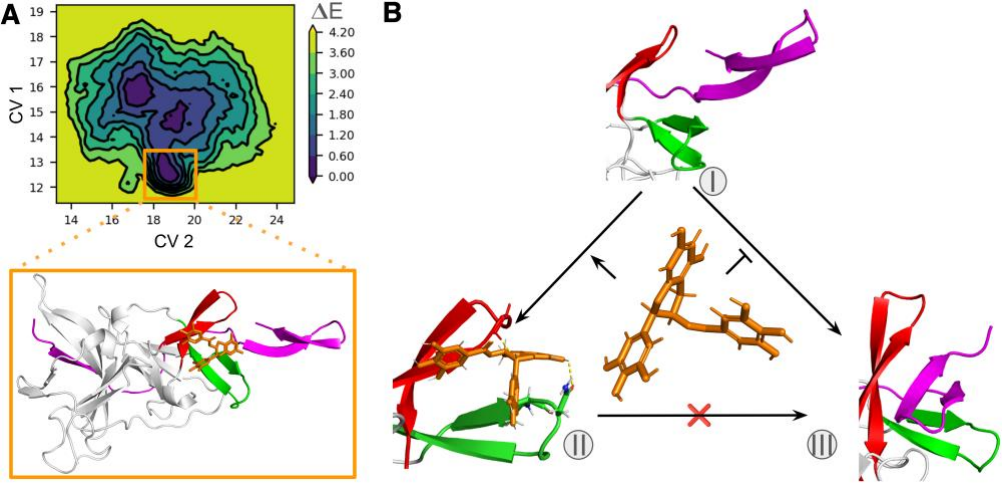
Inhibition of NS2B binding to the claw region by EGCG. A) 2D free energy surface of NS2B binding to the claw region bound to EGCG as obtained by perturbation of two collective variables: CV1 along the center of mass distance between the two loops of the claw and CV2 along the center of mass distance between the claw and the NS2B C-terminal loop. See Fig. S12 for all the replicas. Protein conformations at the two different minima on the energy landscape representing the active and inactive form of dengue NS2B/NS3pro are shown. B) Schematics of the mechanism of NS3pro inhibition by EGCG. Three conformational states of the protease are illustrated: I—inactive open claw conformation; II—inhibitor-bound, tightly closed claw conformation; III—active, NS2B-bound, widely open claw conformation. An allosteric inhibitor such as EGCG stabilizes the tightly closed claw state (I → II) and prevents NS2B binding (I → III). Once EGCG is bound, NS2B cannot displace it (II → III), suggesting a substantial energy barrier.

Finally, we conducted metadynamics analysis to explore NS2B association and dissociation energy profile in presence of EGCG bound within the claw-like structure of T[NT]TG and T[RK][SN]G loop (Fig. 5A). Both the CV1 and CV2, as described earlier, were perturbed simultaneously, to obtain a the free energy landscape of NS2B association and dissociation (CV2) along with claw opening and closing (CV1) in presence of the inhibitor. Figures 5A and S12 show that the energy minima in the free energy landscape shifted from ∼11 Å to more than 15 Å along the CV2 indicating that NS2B can no longer bind into the claw region when EGCG is present. Further, along the CV1 the minima shifts from ∼14 to ∼12 Å suggesting a more closed conformation of the claw in presence of EGCG. Figure 5B schematically illustrates the conformational dynamics. Overall, EGCG restricts the motion of the T[RK][SN]G loop and promotes the claw-like structure towards a more closed conformation, hindering NS2B binding.

## Discussion

Designing effective active site inhibitors for the dengue NS2B/NS3 protease is particularly challenging due to the shallow nature of the active site and the presence of charged residues surrounding it. As a result, more focus has been shifted toward identifying allosteric modulators for this protease. However, most allosteric inhibitors currently exhibit low potency, primarily due to a limited understanding of the underlying allosteric mechanisms. In this study, we use EGCG as an inhibitor model system to provide mechanistic insights into the allosteric regulation of the dengue serine protease.

To investigate the interaction kinetics between EGCG and NS2B/NS3pro, we performed a BLI assay. Our findings reveal that EGCG interacts with the protease in a biphasic manner, indicating two distinct binding events or conformational states. One phase exhibits rapid association with slow dissociation kinetics, suggesting a strong or stable initial interaction, potentially reflecting a high-affinity binding site or a specific conformational state of the protease that facilitates binding. In contrast, the second phase shows slow association and fast dissociation kinetics, indicating a weaker or less stable interaction, possibly involving a different binding site or conformation of the protease with lower affinity for EGCG. We further investigated the thermodynamics of this interaction using an ITC assay. Our ITC data revealed biphasic thermodynamic profiles, with one phase characterized by an endothermic reaction and the other by an exothermic reaction. The endothermic reaction suggests that the binding of EGCG to the protease absorbs heat, indicating an entropically driven interaction, such as displacement of water molecules or conformational changes in the protease. In contrast, the exothermic reaction implies that the binding of EGCG releases heat, reflecting an enthalpically driven interaction, where stable binding interactions, such as hydrogen bonds, van der Waals forces, or electrostatic interactions, contribute to the overall binding affinity. An in vitro enzyme inhibition assay was then performed to examine the nature of inhibition due to EGCG binding the protease inhibits its activity. The results from the enzyme inhibition assay revealed that *v*_max_ gradually decreased, while *K*_M_ remained unchanged. This pattern typically suggests a reduction in the enzyme's catalytic capacity without affecting its affinity for the substrate, indicating that EGCG inhibits the protease in an allosteric manner. Then, we explored the interaction between EGCG and the NS2B/NS3 protease of dengue virus using STD NMR spectroscopy. The results demonstrated a strong interaction between EGCG and the protease, particularly involving the aromatic ring proton groups H2″ and H6″ from the B″ ring, as indicated by the highest STD signals. This suggests that these regions of EGCG play a crucial role in binding to NS2B/NS3pro. Furthermore, the 64% saturation transfer observed for the H2′ and H6′ protons of the B″ aromatic ring reinforces the close proximity of this part of the ligand to the protein. In contrast, the H6 and H8 protons showed significantly weaker STD signals (19%), implying that these protons are more distant from the binding interface. This differential saturation transfer pattern suggests a specific binding orientation of EGCG with NS2B/NS3pro, where the B″ and B′ rings are primarily involved in protein interaction, while other regions contribute less to direct contact. Identification of these interacting functional groups would help to create more potent derivative EGCG by preserving the important contact points with NS2B/NS3pro for sustained functionality and introducing new functional groups to improved specificity and binding efficiency.

To understand how EGCG modulates enzyme activity of the protease, structure and dynamics of the protease were probed using CD spectroscopy and MD simulations. CD data revealed that secondary structural composition of the protease does not change with increasing concentration of EGCG which indicates that ligands may interact with protease at specific sites without altering overall secondary structure of the enzyme. This is a common case where the binding site is pre-formed and does not require large scale conformational adjustments. Some ligands may also interact with protease by side chain contacts or surface interaction. Blind docking simulations followed by further refinements showed that the open claw, which is the NS2B binding site is also the most preferred binding site of EGCG. Finally, the claw opening dynamics under EGCG bound and unbound conditions, in addition to the NS2B biding dynamics into the claw painted an energy landscape that disclosed the intricate mechanism of the NS2B/NS3 activation. The claw when not bound with NS2B or any ligands, can alternate among three different state: a tightly closed state with loop distance of ∼9 Å, a closed sate with loop distance of ∼15 Å and a wide open state with loop distance of ∼20 Å. In a free enzyme, the closed sate with loop distance of ∼15 Å is the most stable where the tightly closed and wide open states are situated at slightly higher energy in the free energy landscape. NS2B binding stabilizes the wide open state of the claw whereas EGCG binding stabilizes the tightly closed state. Inhibitor binding in the claw region, therefore, significantly hinders NS2B binding which leads to reduced enzyme activity.

In conclusion, EGCG shows dengue NS3 protease inhibition by binding to an allosteric site. Binding constant indicates moderately strong interaction, which is enthalpy driven. The binding does not alter the secondary structure of the protein, however, correlated dynamics of the supersecondary structures of the protein gets affected by EGCG binding on the cleft of the claw-like structure made of T[NT]TG and T[RK][SN]G loops. EGCG promotes a more closed state of the claw region, preventing the NS2B binding and thus keeping the enzyme in an inactive state. The plausible mechanism of the allosteric inhibition by EGCG, that was revealed by the metadynamics simulation, suggests future directions in drug design for this family of proteases. More potent derivative of EGCG as well as new molecules can be designed to covalently modify and restrict claw opening thus irreversibly inhibiting the protease.

## Materials and methods

### Materials

The pET-15b plasmid DNA containing wild-type DENV NS2B/NS3pro and the fluorogenic substrate tBOC-GRR-AMC was synthesized by Gencript. Beta-mercaptoethanol, PMSF, EGCG, and EDTA were purchased from Sigma. Luria broth (LB) and IPTG were acquired from Himedia. Tris-HCl, glycerol, glycine, SDS, and ampicillin were sourced from Sisco Research Laboratories. The NI-NTA His-tag column was procured from Cytiva.

### Expression and purification of NS2B-NS3pro

Protease expression and purification were carried out using the similar protocol discussed in our previously published article (28). In brief, the expression vector pET-15b was used for cloning the plasmid DNA of our interest containing the coding sequence for six-histidine-tagged DENV2 wild type NS2B/NS3pro, which was then transformed into *Escherichia coli* BL21 bacterial cells. The transformed cells were cultured in LB media supplemented with 100 µg/ml ampicillin at 37°C. Upon reaching an OD600 of 0.6, the cells were induced with 1 mM IPTG and further incubated at 18°C for 16 h with agitation at 180 rpm. Subsequently, the cells were harvested by centrifugation at 6,000 rpm for 15 min at 4°C and lysed using a lysis buffer consisting of 10 mM PMSF, 50 mM Tris pH 8.5, 5% glycerol, 2 mM imidazole, 50 mM NaCl, and 5 mM β-mercaptoethanol. After centrifugation at 10,000 rpm for 55 min at 4°C, the resulting cell lysate was applied to a 5 ml NI-NTA column for affinity purification using the ÄKTA-Start FPLC system. The His-tagged protein was eluted from the column using an elution buffer containing 100 mM imidazole, 30 mM NaCl, and 100 mM Tris-HCl pH 8.7. The fractions containing the purified protein were dialyzed and concentrated. The purity of the purified protein was confirmed by SDS-PAGE and MALDI-TOF spectrometry.

### Enzyme inhibition assay

The enzyme inhibition assay was conducted by adapting the similar protocol published in our previous research article (28). Briefly, in a reaction mixture of 100 μl, consisting of 100 mM Tris-HCl buffer at pH 9, 300 nM NS2B/NS3pro, and varying concentrations of EGCG, the components were pre-incubated at 37°C for 30 min. A control sample lacking EGCG was also prepared. The reaction was initiated by the addition of fluorogenic peptide tBOC-GRR-AMC and further incubated at 37°C for an additional 30 min. This experiment was repeated three times. Fluorescence intensity was measured using an Agilent Cary Eclipse Fluorescence spectrophotometer, with excitation and emission wavelengths set at 365 and 439 nm, respectively. Michaelis–Menten and Lineweaver-Burk plots were generated using wolfram mathematica 13.0, employing the Eqs. 1 and 2 as given below:


(1) $$v=\frac{v_{\max}[S]}{K_{M}+[S]}$$

(2) $$v=\frac{K_{M}}{v_{\max}[S]}+\frac{1}{v_{\max}},$$

where *v* represents the reaction velocity, *v*_max_ denotes maximum velocity at saturated substrate concentration, *K_M_* is the Michaelis constant, which represents the substrate concentration at which the reaction velocity is half of *v*_max_ and [*S*] is substrate concentration. IC_50_ value was calculated by fitting *v*_max_ versus [EGCG] using Eq. 3 as given below:


(3) $$N_{i}=N_{0}2^{\left(i/IC_{50}\right)},$$

where *N_i_* represents enzyme activity at a given inhibitor concentration *i* and *N*_0_ denotes enzyme activity without inhibitor. IC_50_ is inhibitor concentration at which enzyme activity reduces to 50%. All data were processed using Wolfram Mathematica 13.0.

### Isothermal titration calorimetry

The binding thermodynamics between dengue NS2B/NS3pro and EGCG were investigated using a Nano-ITC instrument at 310 K. Both the protease and EGCG were dissolved in a 10 mM sodium phosphate buffer of pH 7.4 and degassed prior to the experiment. A concentration of 100 μM EGCG was loaded into the syringe and titrated against 10 μM NS2B/NS3pro in the sample cell. A minimum of 30 injections were made against NS2B/NS3pro, each containing 100 μM EGCG. A control was performed by injecting a 10 mM sodium phosphate buffer. During the injection, the reference cell contained water. The sample cell was rotated at 200 RPM for proper mixing. Data analysis was done with Wolfram Mathematica 13.0 using biphasic ITC equation (Eq. 4):


(4) $$\Delta Q=A_{\min}+\frac{A_{\max1}-A_{\min}}{1+10^{(x-n_{1})k_{1}}}+\frac{A_{\max2}-A_{\min}}{1+10^{(n_{2}-x)k_{2}}},$$

where Δ*Q* represents observed enthalpy change, (*A*_max1_ − *A*_min_) corresponds to enthalpy change of endothermic process (Δ*H*_1_) and (*A*_max2_ − *A*_min_) represents the enthalpy change of exothermic process (Δ*H*_2_). *n*_1_ and *n*_2_ denote stoichiometry of endothermic and exothermic processes respectively while *k*_1_ and *k*_2_ are rate constants for endothermic and exothermic reaction respectively. Equilibrium constant (*K_a_*) for both of the reactions was determined by taking a derivative of Δ*Q* with respect to mole ratio (Fig. S5). Δ*G* was calculated from the Eq. 5.


(5) $$\Delta G=-\mathrm{RTln}(K_{a}),$$

where *R* is the universal gas constant and *T* is the temperature in Kelvin and *K_a_* is the equilibrium constant. Entropy changes (Δ*S*) was calculated by using the Eq. 6.


(6) ΔG=ΔH−TΔS.

### Biolayer interferometry

The biolayer interferometry experiment was conducted using the Octet RED96 instrument (FortéBio) with Sartorius Ni-NTA biosensors. Prior to the kinetic experiment, the Ni-NTA biosensors were hydrated for 10 min in 96-well plates. The binding assay commenced by positioning the biosensors on the reading tip of the BLI instrument. The entire kinetics experiment was conducted in a 20 mM NaP buffer with a pH of 7.4. Initially, the biosensors were incubated in the 20 mM NaP buffer for 30 s to establish a baseline. Subsequently, in the loading step, the biosensors were transferred to a solution containing 25 µM His-tagged NS2B/NS3pro protein for 120 s. Following the loading step, the biosensors were returned to the assay buffer for baseline stabilization for an additional 120 s. To investigate the interaction between NS2B/NS3pro and EGCG, the biosensors were transferred to solutions containing different concentrations of EGCG (0, 25, 50, and 100 µM) until equilibrium was attained (500 s). Further transfer to Nap buffer for dissociation (500 s). Subsequently, dissociation of the whole complex was initiated by shifting the biosensors into a Glycine buffer with a pH of 2.7. The entire kinetics assay was conducted at a temperature of 25°C. All data were analyzed using Wolfram Mathematica 13.0. Response (R) was appropriately fitted in 2:1 association and dissociation kinetics equation (Eq. 7):


(7) $$R=\{\begin{array}{cc} R_{\max1}(1-e^{-k_{a1}t})+R_{\max2}(1-e^{-k_{a2}t}), & t\leq t_{n}\\ R_{\max1}e^{-k_{d1}(t-t_{n})}+R_{\max2}e^{-k_{d2}(t-t_{n})}, & t>t_{n} \end{array}$$

where *R* is biolayer interferometry response; *R*_max1_ and *R*_max2_ denote maximum response for the first and second ligand binding for a two site binding model, respectively. *k_a_*_1_ and *k_a_*_2_ is the association rate constant of the two ligands, respectively while *k_d_*_1_ and *k_d_*_2_ represent the dissociation rate constant of the two ligands and t denotes time in seconds, *t_n_* is the association reaction time (500 s in this case).

### CD spectroscopy

We used BioLogic MOS500 CD spectrophotometer to study change in secondary structure and melting temperature shifting of NS2B/NS3pro after binding with EGCG. To study change secondary structure of protease we titrate 10 µM NS2B/NS3pro with different concentrations (10, 25, and 50 µM) of EGCG in 10 mM NaP buffer. 190–250 nm spectra were taken with a pathlength of 1 mm after subtracting buffer background along with respective concentration of EGCG. We calculate the percentage of secondary structure of protease using our previously published protocol (44), and the fitted peaks are shown in Fig. S6.

### STD NMR spectroscopy

The 3 mM EGCG sample was dissolved in the 100% deuterated phosphate buffer solution with a pH of 7.2. The stock solution of NS2B/NS3pro protein was also prepared in the same buffer solution with the same pH of 7.2. All NMR spectra were recorded on a Bruker Avance III 500 MHz spectrometer, equipped with a 5 mm RT probe at 298 K using standard STD pulse sequences and excitation sculpting was used for water suppression. Data acquisition and processing were performed with Topspin 3.1 software (Bruker).

STD NMR spectra were collected at a 1:100 NS2B/NS3pro/EGCG mixture ratio. A sequence of Gaussian-shaped pulses with a 1% truncation, each 49 ms in duration, and separated by a 1 ms delay were used to selectively irradiate NS2B/NS3pro. A total of 40 chosen pulses were used, resulting in a total saturation duration of 2 s. The on-resonance for NS2B/NS3pro was set to −1.0 ppm, and the off-resonance was set at 40 ppm, where neither the protein nor the EGCG resonances were present. The difference spectrum, which incorporates signals coming from the saturation transfer, is obtained by subtracting the two spectra (on-resonance and off-resonance) by phase cycling. The reference spectrum was recorded with 1,024 scans, while the difference spectrum was obtained with 2,048 scans. In order to identify the group epitope mapping, seven saturation times (*T*_sat_): 0.5, 1.0, 1.5, 2.0, 2.5, 3.0, and 5.0 s were chosen to generate the STD build-up curves using the monoexponential equation (Eq. 8):


(8) $$\mathrm{ST}D_{\mathrm{amplification}\;\mathrm{factor}}=\mathrm{ST}D_{\max}(1-e^{k_{st}t}),$$

where STD_max_ is the maximal STD intensity and *k_st_* is the saturation rate constant for EGCG (3 mM) in the presence of NS2B/NS3pro at zero saturation time.

### Molecular docking

Docking of EGCG on NS2B/NS3pro was performed following a previously published protocol (45). In brief, AutoDock 4.2 (46) was used to generate an ensemble of docked conformers under blind-docking setup where the whole protein was included in the search space. The protein structure information was obtained from the protein data bank (PDB ID 4K9M and 3U1I). The EGCG structure was obtained from PubChem (CID: 65064). Prior to docking, the protein structure was prepared by removing all the heteroatoms, crystal waters, alternate atom positions, and filling any missing side chain atoms. Both the protein and the ligand structures were minimized using Schrodinger Maestro (Academic release 2020-4) prior to docking. Genetic algorithm was used in AutoDock 4.2, and it was run 100 times to generate a statistically significant number of docked poses. Each genetic algorithm run was set to terminate after 25 million energy evaluations or 27,000 generations. Spatial deviation of the conformers (RMSD) and their binding energies were then distributed on a smooth density histogram using Wolfram Mathematica 12.0. High density and low energy clusters were identified and further targeted docking was performed restricting the search space to the most probable binding site.

### Molecular dynamics simulations

The Desmond molecular dynamics program as implemented in the Schrodinger Maestro (Academic release 2020-4) was used to perform molecular dynamics simulation (47). A previously published protocol was followed (28). Prior to MD simulation, the protein (PDB ID 4K9M and 3U1I) was processed by filling up missing side chains, adding hydrogens, assigning correct bond orders and the forcefield specific atom types. Protonation states and the hydrogen bonding networks of amino acid residues were optimized for pH 7.4 using the PROPKA method (48) during the protein preparation stage as implemented in Schrodinger Maestro. An cubic periodic boundary box was defined and the protein was placed at the center of the box such that there were at least 10 Å buffer regions on each side of the protein. Thus, the periodic images of the protein were at least 20 Å apart from each other. The box sizes were approximately 90 Å × 90 Å × 90 Å. Preoptimized simple point charge (SPC) water model (49) was used to entirely fill the simulation box. Sodium ions (Na^+^) were used to neutralize charges. MD was run in optimized potentials for liquid simulations force field (50, 51). The simulations were conducted using either the canonical (NVT) or isothermal–isobaric (NPT) ensemble. Desmond employs the M-SHAKE algorithm to constrain all high-frequency X–H bonds. A four-step relaxation protocol was employed to equilibrate the system prior to the production run. The process began with 100 ps of Brownian dynamics in the NVT ensemble at 10 K, with positional restraints applied to solute heavy atoms. This was followed by 12 ps of Langevin dynamics under the same ensemble (NVT, 10 K), also with solute heavy atom restraints. Subsequently, 12 ps of Langevin dynamics was performed in the NPT ensemble at 10 K and 1.01235 bar, with restraints maintained. Finally, the system was heated to physiological conditions with 12 ps of Langevin dynamics in the NPT ensemble at 300 K and 1.01235 bar, still under restraints (28). Following equilibration, production molecular dynamics simulations were carried out for 250 ns in the NPT ensemble at 300 K and 1.01235 bar, without restraints on the solute heavy atoms. A reference system propagator algorithm integrator was used with near, far, and out time steps of 2, 2, and 6 fs, respectively (52). The isotropic pressure was applied using the Martyna–Tobias–Klein barostat method with relaxation time of 2 ps. The Nose–Hoover chain thermostat method was used with relaxation time of 1 ps. Long-range coulombic interaction was treated with the particle mesh Ewald method. For short-range coulombic interactions, the cutoff radius was 9 Å. Simulation of NS2B/NS3pro in presence of EGCG at different mole ratios as well as the EGCG docked complexes were performed following the same protocol discussed above. The mole ratios were approximated from the number ratio of the two solutes. 1, 3, 10, 32, and 100 EGCG molecules were placed randomly in the simulation box and the rest of the volume was filled with preoptimized SPC water model to obtain 1:1, 1:3, 1:10, 1:32, and 1:100 molar ratios of the inhibitor. Blind docking of EGCG with NS2B/NS3pro was done using AutoDock 4.2 and the best docked complexes were used for MD simulation. Schrodinger Maestro and the VMD timeline tools were used to analyze trajectories. Loop distances were calculated from the trajectories (Fig. S7). Root mean square deviations (RMSD) are given in Fig. S8.

### Metadynamics simulations

For metadynamics simulations, the systems were prepared and equilibrated as described in the previous section. Additionally, collective variables were defined and a biasing potential was added using the metadynamics module in Desmond (Schrodinger Maestro). The collective variable is defined as the distance between the center of mass of the T[RK][SN]G loop backbone and the center of mass of T[NT]TG loop backbone with wall at 25 Å. Gaussian potentials of 0.01 kcal/mol height were injected at an interval of 0.09 ps. For 2D scan, another collective variable is defined as the distance between the center of mass of the NS2B hairpin loop backbone and the center of the claw region. The relaxation time of the biasing potential was increased to 0.2 ps from 0.09 ps. Each simulation was run for 40 ns in the NPT ensemble at 300 K and 1.01235 bar. The claw opening dynamics for free protein, EGCG bound, or NS2B bound conditions were repeated 10 times with different random seeds in order to achieve convergence. Wang et al showed that the free energy profile converges when a sufficient number of replicas are averaged (53). The free energy profiles reported here represent the average of 10 independent runs. The 2D scans were repeated 10 times. The primitive profiles are included in the Figs. S9–S12.

### Normal mode analysis

Normal modes were calculated using the NMWizard plugin in VMD (Visual Molecular Dynamics, version 1.9.4a51), which interfaces with ProDy (version 1.10.10). Anisotropic network model method was applied to calculate the dominant motions (54). The MD trajectories of the protein in different mole ratios of inhibitor were used. Prior to the normal mode calculations, protein backbone atoms (C, N, and Cα) were extracted from the MD trajectories and aligned to the first frame to remove global translational and rotational motions. The elastic network model was constructed considering default ProDy parameters with a cutoff distance of 15 Å, and uniform spring constants were applied between all residue pairs within the cutoff. For each system, the first 10 nontrivial modes were computed. The mobility plots were generated from the first nontrivial mode (mode 1), which captured the dominant collective motion for the system. No normalization or scaling was applied to the resulting fluctuations. Per-residue mobilities reflect the predicted amplitude of motion for each *C*_α_ atom along the first mode.

## Supplementary Material

pgaf276_Supplementary_Data

## Data Availability

All relevant data supporting this study are provided in the Supplementary material.
