# Supplementary material for: Dynamic claw of dengue protease unveils druggability potential with high affinity allosteric inhibitors
Source: PNAS Nexus. 2025 Nov 4;4(11):pgaf276. doi: 10.1093/pnasnexus/pgaf276 (PMC12586334; doi:10.1093/pnasnexus/pgaf276)
Supplement: pgaf276_Supplementary_Data [file pgaf276_supplementary_data.docx]

**Supplementary Material for**

Dynamic Claw of Dengue Protease Unveils Druggability Potential with High Affinity Allosteric Inhibitors

Mrinmay Bhunia^1,2^, Rajdip Misra^1^, Anupam Maity^1,2^, Sk Abdul Mohid^3^, Shubham Kundu^1^, Anirban Bhunia^3^, Nakul C. Maiti^1,2,^*, Uttam Pal^1,^*

1 Structural Biology and Bioinformatics Division, Indian Institute of Chemical Biology, Council of Scientific and Industrial Research, 4, Raja S.C. Mullick Road, Kolkata 700032, India.

2 Academy of Scientific and Innovative Research (AcSIR), CSIR-Human Resource Development Centre, Ghaziabad, Uttar Pradesh, 201002, India.

3 Chemical Sciences, Unified Academic Campus, Bose Institute, EN-80, Sector V, Bidhan Nagar, Kolkata 700091, India.

*Nakul C. Maiti, Uttam Pal

**Email:**  ncmaiti@iicb.res.in, uttam2707@gmail.com

**Author Contributions:** M.B., N.C.M., and U.P. designed research; M.B., R.M., A.M., S.A.M., and S.K. performed research; M.B., A.B., N.C.M. and U.P. analyzed data; M.B., N.C.M., and U.P wrote the paper.

**Competing Interest Statement:** The authors declare no competing interest.

**Classification:** Physical Sciences: Biophysics and Computational Biology

**Keywords:** Loop dynamics, conformational switching, free energy landscape, enzyme activity.

**This PDF file includes:**

Supplementary Material


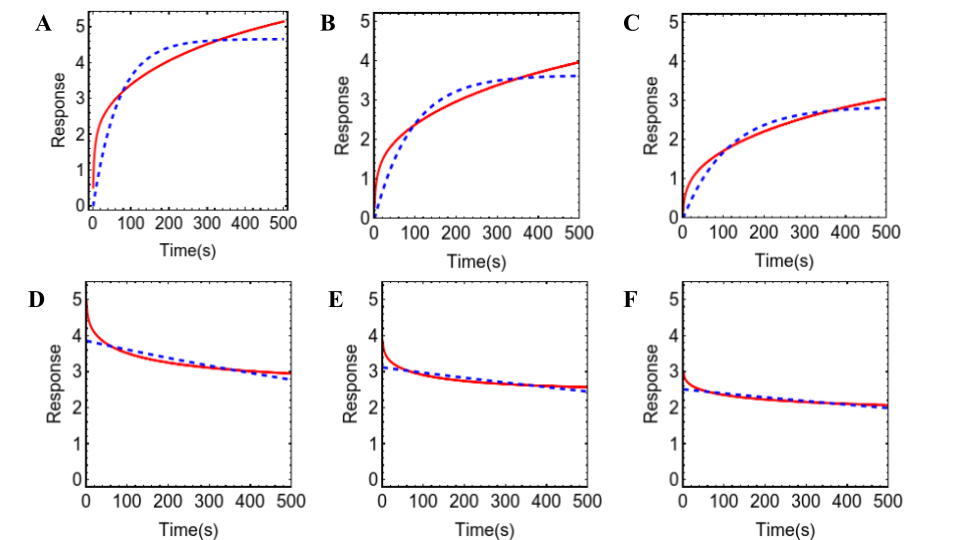


**Figure S1.** Biolayer interferometry sensogram: (A–C) 1:1 model fit of the BLI association curves for 100 µM, 50 µM, and 25 µM EGCG, respectively, showing suboptimal fitting to this model. (D–F) 1:1 model dissociation curves for 100 µM, 50 µM, and 25 µM EGCG, respectively.

**Figure S2.** Biolayer interferometry sensogram: A-B) 2:1 model fit and deconvolution of the association curves for 50 µM and 25 µM EGCG, respectively, demonstrating a close fit of the data to the model. C-D) 2:1 model fit and deconvolution of the dissociation curves for 50 µM and 25 µM EGCG, respectively, confirming that the data are closely aligned with the model.


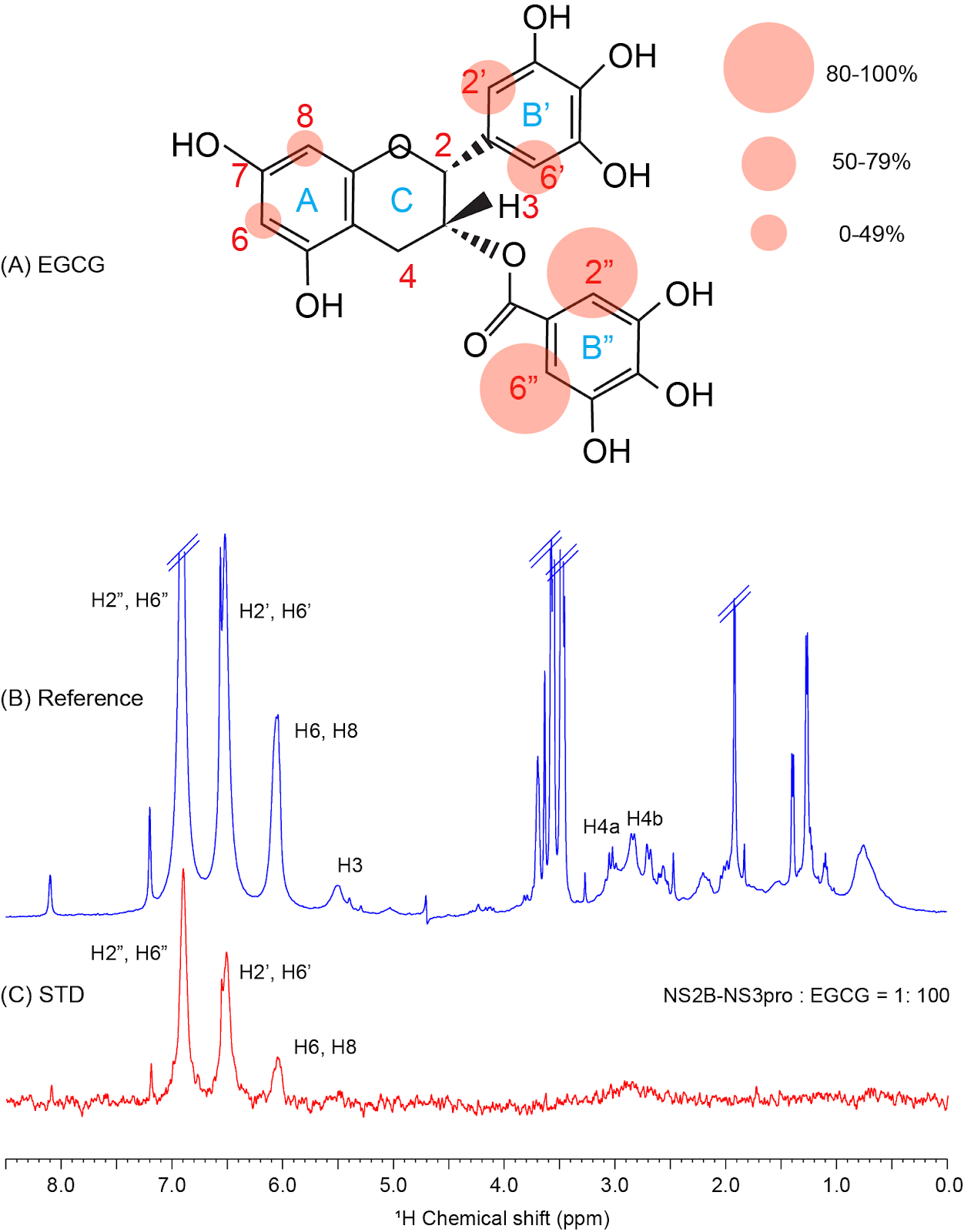


**Figure S3.** Functional group epitope mapping of EGCG (A) Chemical structure and group epitope mapping of the EGCG molecule. 1D STD NMR analysis of reference (B) and STD (C) spectrum of EGCG compound in the presence of NS2B-NS3pro. The aromatic ring protons of B’ and B” rings are in close proximity to the NS2B-NS3pro as indicated by the strong peak intensity in the STD spectrum. The H6, H8 protons showed weak STD signals (19%) which indicates that they are loosely bound to the NS2B-NS3pro. To reflect the proximity of protons to the binding of NS2B-NS3pro, three different-sized circles were used. Larger saturation transfers (80–100%) show that the ligand protons are near the protein, whereas medium and smaller transfers (50-79% and 0–49%) show that the ligand protons are far away from the protein.


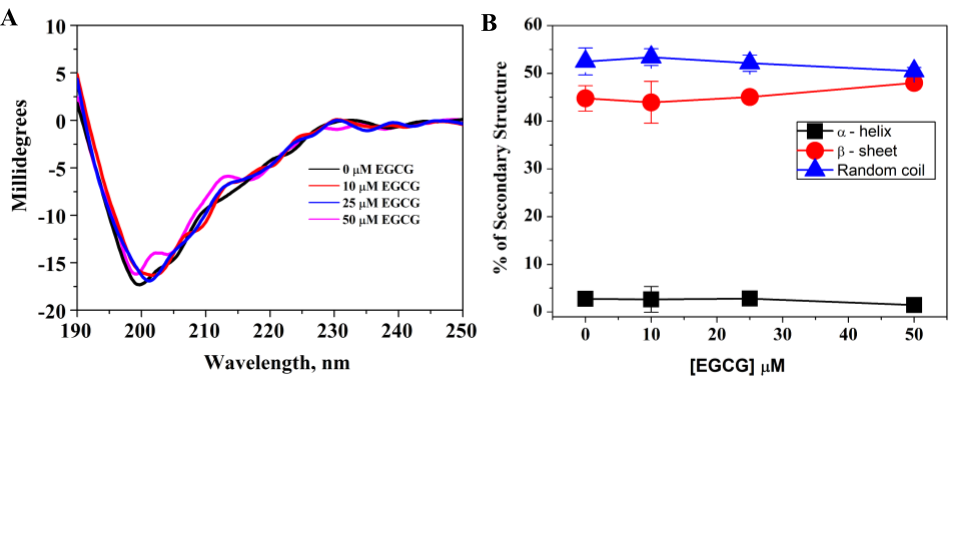


**Figure S4.** NS2B/NS3pro secondary structural changes upon EGCG binding. (A) CD spectra of NS2B/NS3pro at various EGCG concentrations, showing that the protease is predominantly composed of random coils and β-sheets. (B) Deconvolution of the CD spectra reveals the percentage of secondary structure as a function of EGCG concentration, indicating that no significant structural alterations occur with increasing EGCG concentration.


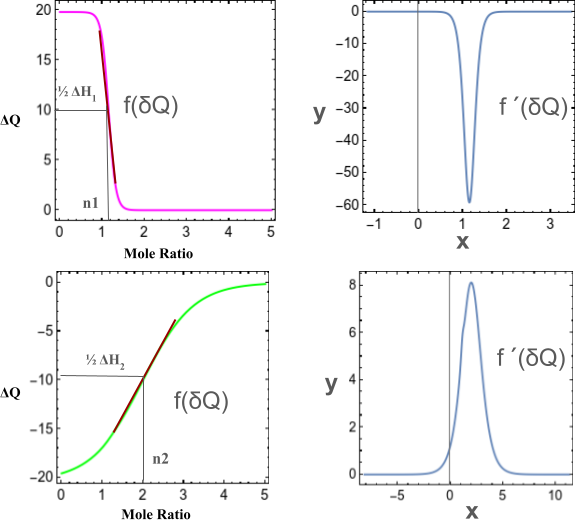


**Figure S5.** Fitted exotherm and endotherm of ITC data ( left panel). Corresponding derivative of the fitted functions.


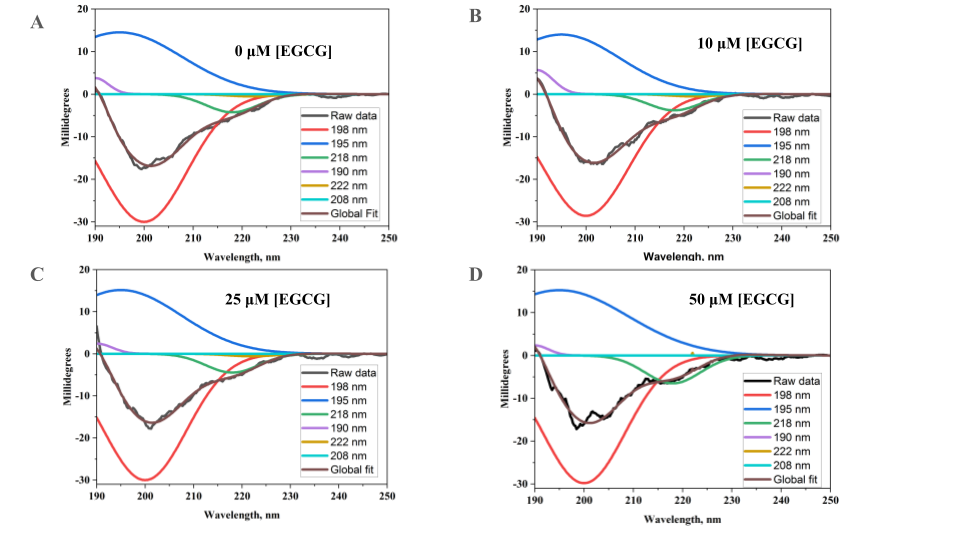


**Figure S6.** Deconvolution of circular dichroism spectroscopy data for dengue NS2B/NS3pro at varying EGCG concentrations (0 µM, 10 µM, 25 µM, and 50 µM), illustrating changes in the secondary structural composition of the protease upon EGCG binding.


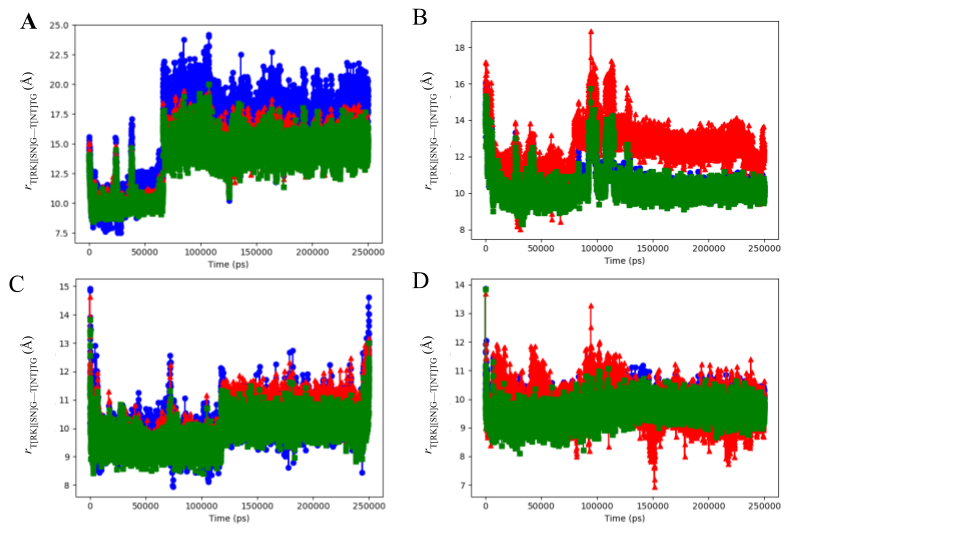


**Figure S7.** Loop distance at different mole ratios of EGCG. A) EGCG:NS2B/NS3pro::3:1 B) EGCG:NS2B/NS3pro::10:1 C) EGCG:NS2B/NS3pro::32:1 D) EGCG:NS2B/NS3pro::100:1. Cα distances of Arg213—Asn175 (blue), Ala216—Thr174 (red), and Thr212—Gly177 (green) are shown as a function of time.


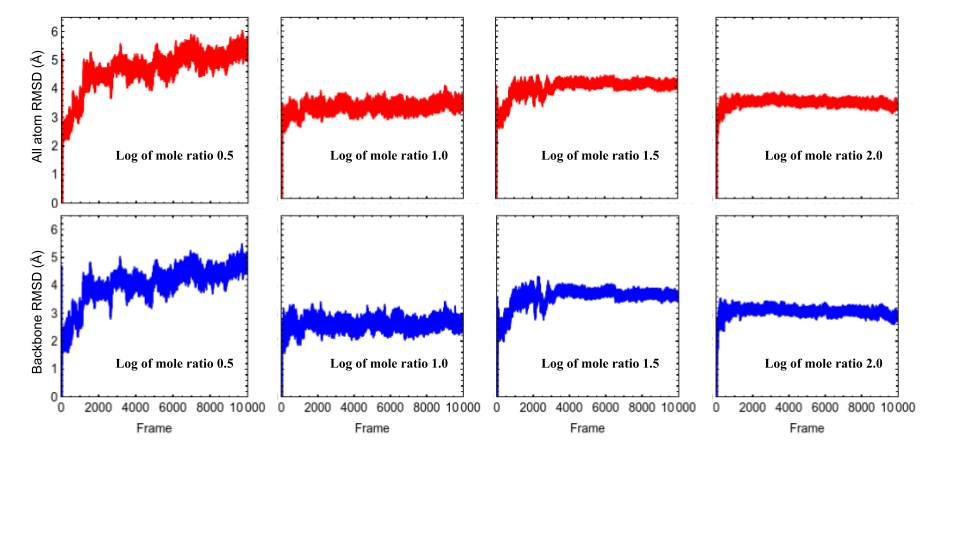


**Figure S8.** RMSD profiles. (Top panel) All atom RMSD of NS2B/NS3pro at different molar ratios of EGCG. (Bottom Panel) Backbone RMSD of NS2B/NS3pro at different molar ratios of EGCG. Simulation time 250ns.


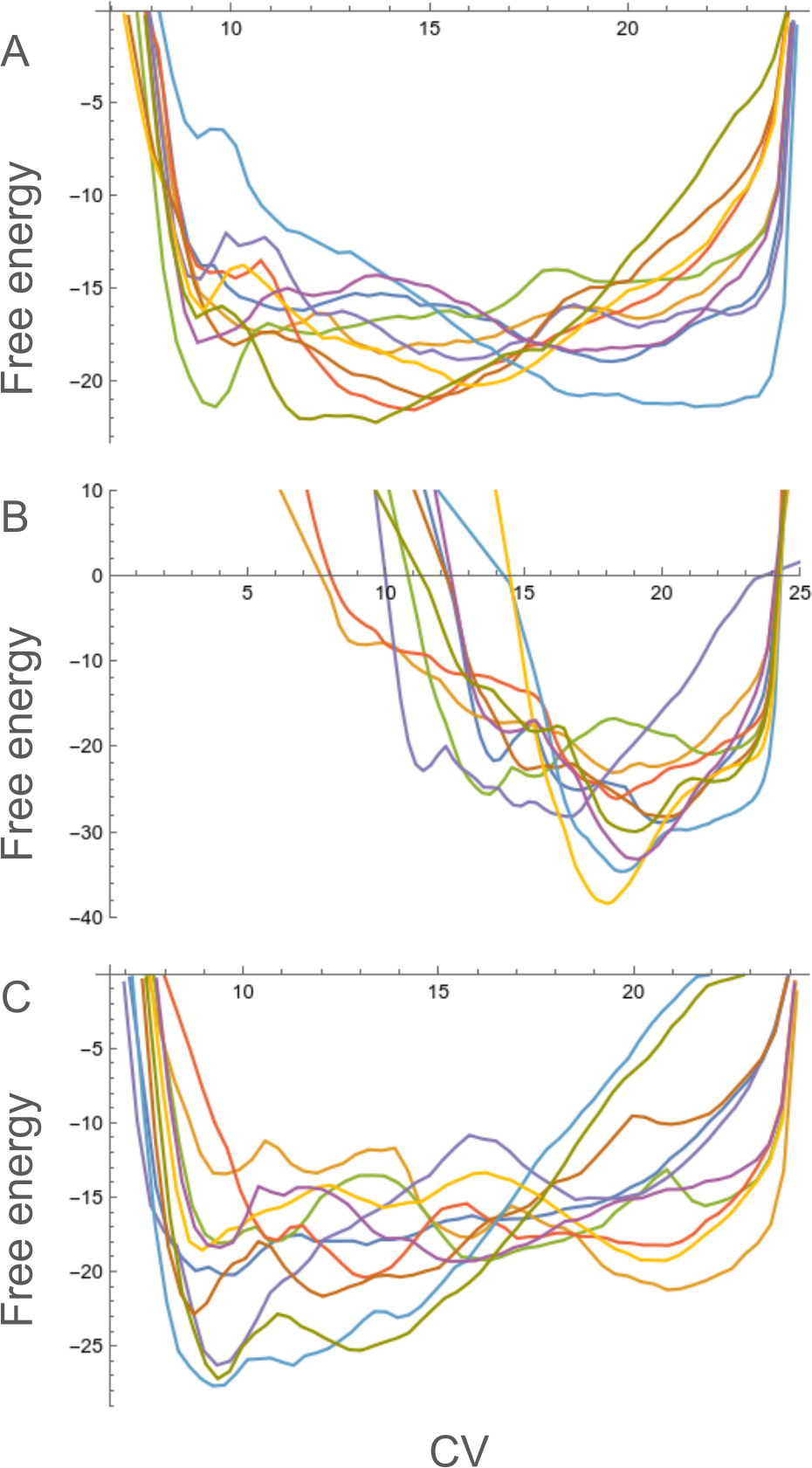


**Figure S9. Primitive free energy profiles for claw opening dynamics.** (A) Free protein in inactive conformation. (B) Free protein in active conformation. (C) EGCG bound conformation. The collective variable (CV) is defined as the distance between the center of mass of T[NT]TG and T[RK][SN]G loops. The free energy values are in kcal/mol.


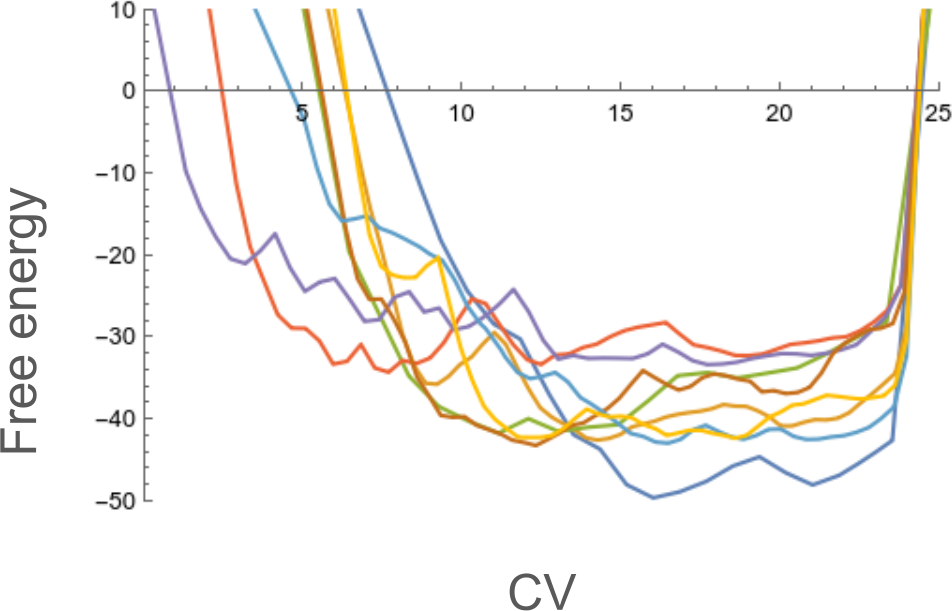


**Figure S10. Primitive free energy profiles for NS2B binding into the Claw.** The collective variable (CV) is defined as the distance between the NS2B and the center of mass of claw region (T[NT]TG and T[RK][SN]G loops together). The free energy values are in kcal/mol.


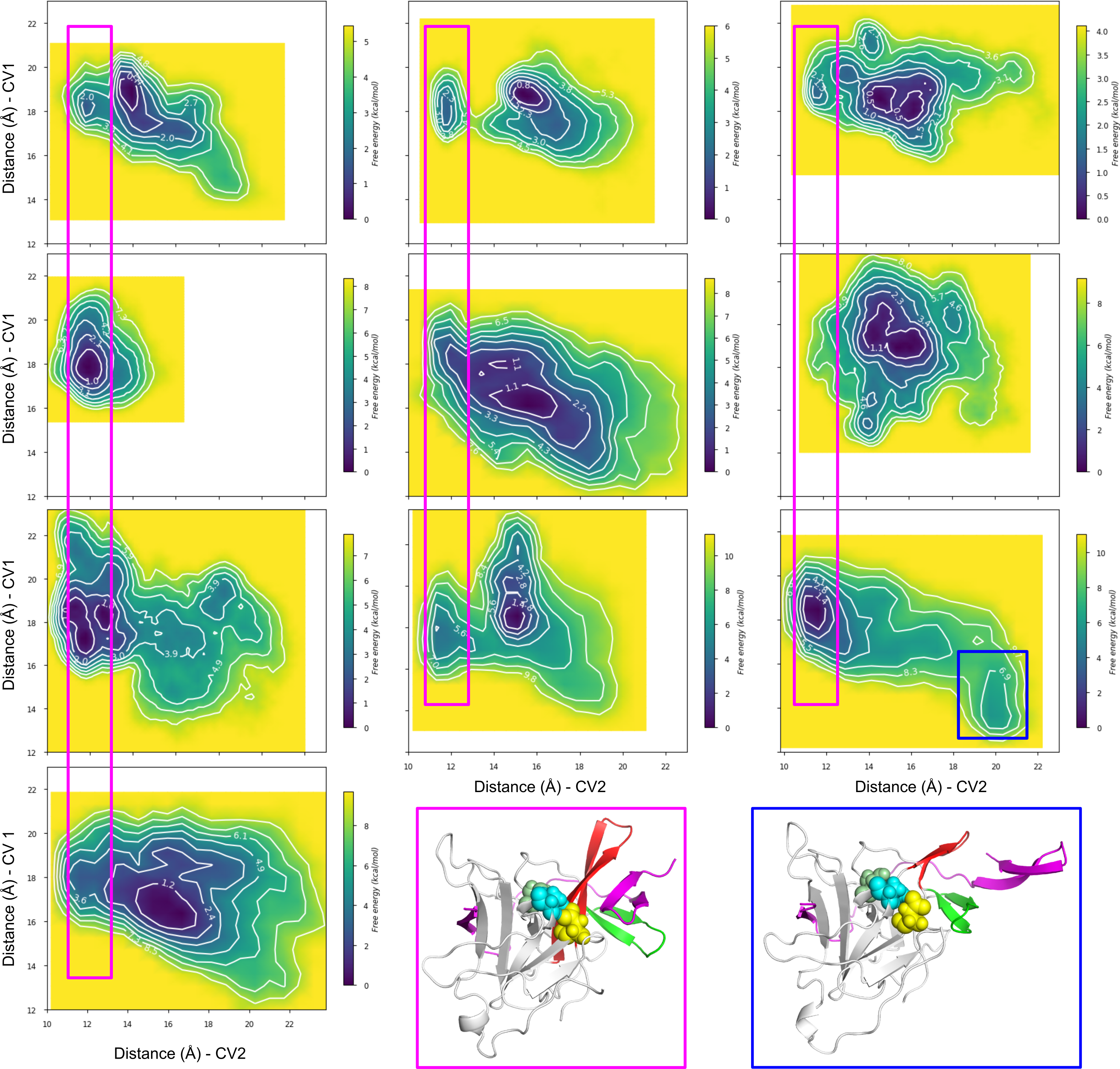


**Figure S11. Primitive free energy profiles for NS2B binding into the Claw.** The claw opening dynamics was also perturbed with bias potential. The collective variable (CV1) is defined the distance between the NS2B and the center of mass of claw region (T[NT]TG and T[RK][SN]G loops together). The CV2 is defined as the distance between the center of mass of T[NT]TG and T[RK][SN]G loops. The regions where NS2B in inserted into the claw is marked with magenta rectangle. In all the ten replicas the NS2B bound active conformations could be observed. Representative structure of NS2B bound and unbound conformations are shown outlined in magenta and blue rectangles, respectively.


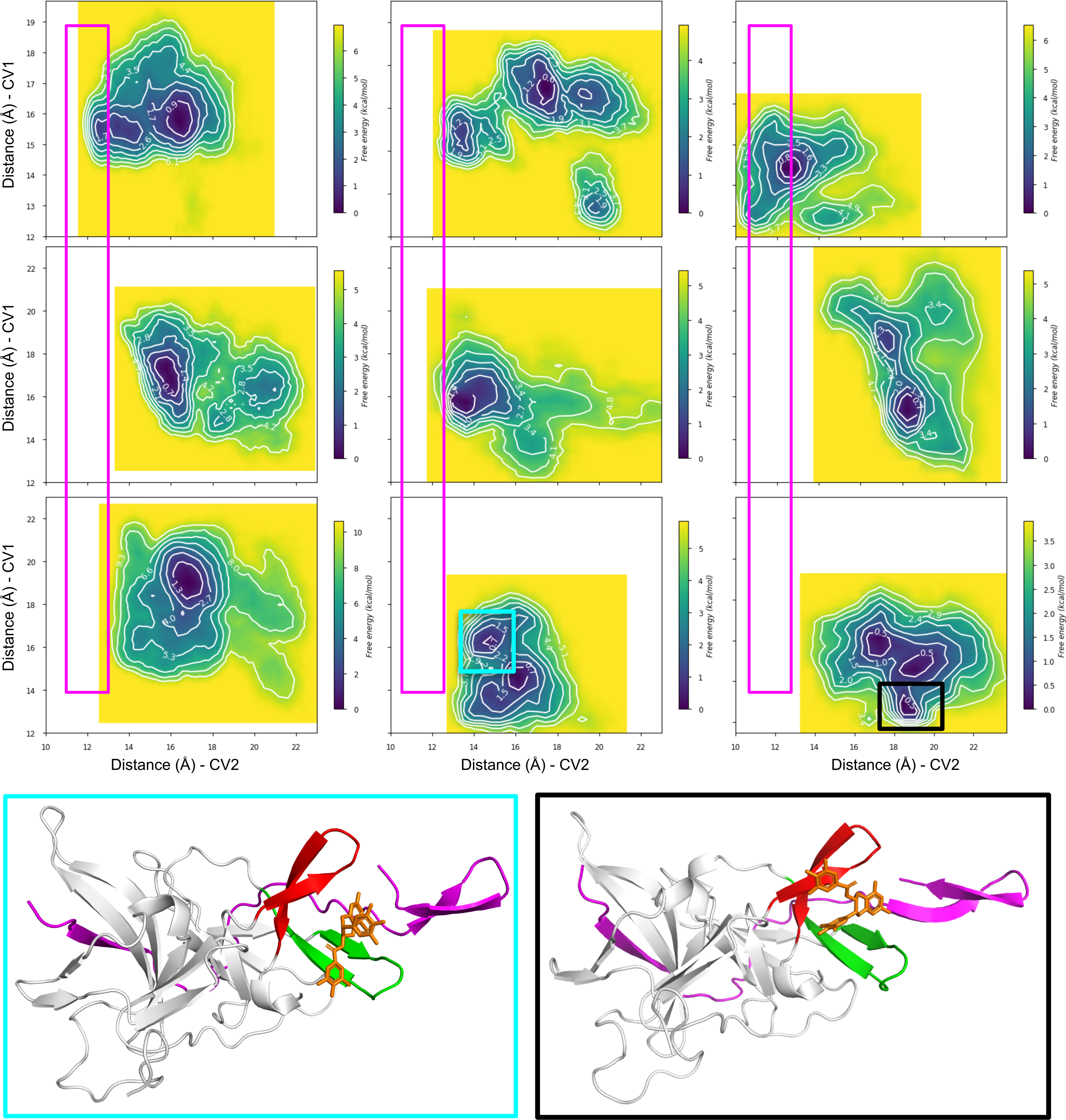


**Figure S12. Primitive free energy profiles for NS2B binding into the Claw in presence of EGCG bound into the claw region.** The claw opening dynamics was also perturbed with bias potential. The collective variable (CV1) is defined the distance between the NS2B and the center of mass of claw region (T[NT]TG and T[RK][SN]G loops together). The CV2 is defined as the distance between the center of mass of T[NT]TG and T[RK][SN]G loops. Out of the ten replicas, only in one instance (top right profile), NS2B was found to bind into the claw region displacing EGCG. Representative structures show the relative position of NS2B with respect to the claw region bound to EGCG.
